# Deep-learning method for fully automatic segmentation of the abdominal aortic aneurysm from computed tomography imaging

**DOI:** 10.3389/fcvm.2022.1040053

**Published:** 2023-01-05

**Authors:** Atefeh Abdolmanafi, Arianna Forneris, Randy D. Moore, Elena S. Di Martino

**Affiliations:** ^1^R&D Department, ViTAA Medical Solutions, Montreal, QC, Canada; ^2^Department of Biomedical Engineering, Schulich School of Engineering, University of Calgary, Calgary, AB, Canada; ^3^Division of Vascular Surgery, University of Calgary, Calgary, AB, Canada

**Keywords:** abdominal aortic aneurysm, computed tomography imaging, deep learning, medical image analysis, tissue characterization

## Abstract

Abdominal aortic aneurysm (AAA) is one of the leading causes of death worldwide. AAAs often remain asymptomatic until they are either close to rupturing or they cause pressure to the spine and/or other organs. Fast progression has been linked to future clinical outcomes. Therefore, a reliable and efficient system to quantify geometric properties and growth will enable better clinical prognoses for aneurysms. Different imaging systems can be used to locate and characterize an aneurysm; computed tomography (CT) is the modality of choice in many clinical centers to monitor later stages of the disease and plan surgical treatment. The lack of accurate and automated techniques to segment the outer wall and lumen of the aneurysm results in either simplified measurements that focus on few salient features or time-consuming segmentation affected by high inter- and intra-operator variability. To overcome these limitations, we propose a model for segmenting AAA tissues automatically by using a trained deep learning-based approach. The model is composed of three different steps starting with the extraction of the aorta and iliac arteries followed by the detection of the lumen and other AAA tissues. The results of the automated segmentation demonstrate very good agreement when compared to manual segmentation performed by an expert.

## 1. Introduction

Abdominal aortic aneurysm (AAA) is defined as a focal dilation of the aorta where the maximum diameter exceeds the normal diameter by at least 1.5 times (1). When not diagnosed and treated, an aneurysm may continue to enlarge until it ruptures, resulting in significant mortality and morbidity (2). AAA is accompanied by the alteration of the major structural proteins (elastin and collagen) in the aortic wall, which results in irreversible enlargement and loss of structural integrity. The majority of aneurysms are characterized by the presence of intraluminal thrombus (ILT), which is associated with hypoxia and is a locus of inflammatory processes that contribute to arterial wall weakening and growth (1–3). Calcification is often present in AAAs and is recognized as one of the factors contributing to the progression of aneurysmal disease through local stiffening and stress concentration (4, 5).

**Figure 1:**
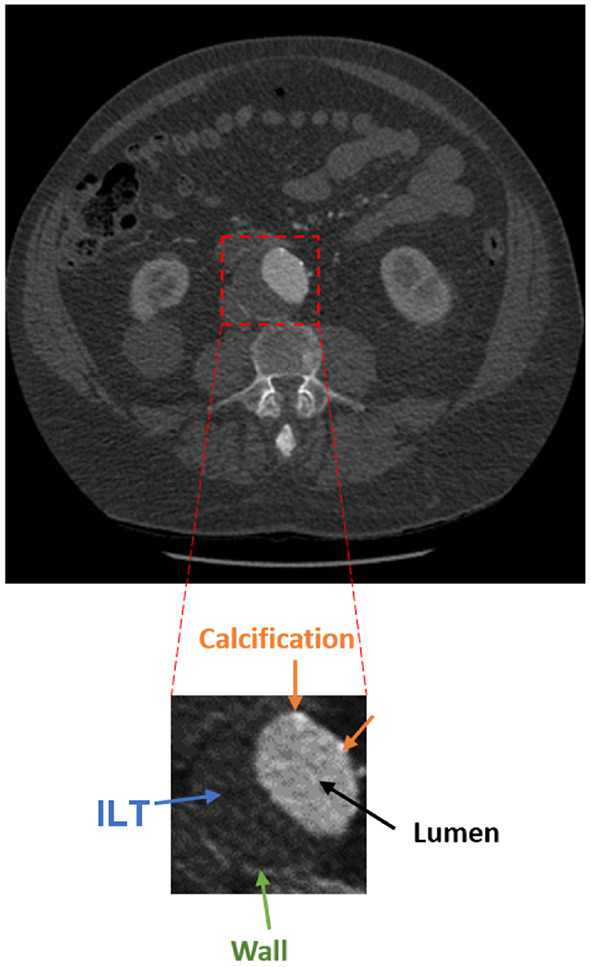
Axial acquisition of an abdominal CT scan showing an AAA. The aorta is a small fraction of the whole image and is composed of the lumen (black), ILT (blue), wall (green), and calcification (orange).

The presence of an aneurysm and its progression are normally assessed using CT imaging. Figure 1 shows an axial image from a CT scan of the abdomen of a patient. The aorta, a small fraction of the whole image, is located anteriorly to the spinal column and is composed of the wall, lumen, ILT, and calcification. The adoption of a three-dimensional assessment of the aorta is limited by the lack of standardized automated segmentation tools for the aorta, in particular for the aortic wall (6).

Several traditional machine-learning methods have been proposed to segment the different surfaces and volumes comprising the aneurysm, including the wall-to-lumen/thrombus interface and the lumen volume. Applications have included graph cut theory (7, 8), intensity- and gradient-based segmentation approaches (9), and variable neighborhood search (10). Many studies have focused on identifying the ILT as the wall-to-ILT surface is difficult to segment due to similarities in the image intensity with neighboring structures. The ILT was segmented using a trained deep convolutional neural network (CNN) on post-operative CT images by López-Linares et al. (11). A level set method was applied to detect the ILT and the outer wall boundary by Zohios et al. (12). A segmentation of the lumen and ILT was also achieved using an active contour approach by Lareyre et al. (13). A semi-automatic interactive image segmentation method was proposed by Maiora et al. (14) to detect the aorta. In this approach, the gray level co-occurrence matrix (GLCM) and the local binary patterns were used as features to train a random forest classifier. An intensity-based approach followed by neural networks was used to detect the lumen and wall contours by Shum et al. (15). Another contour-based segmentation approach to extract the aorta was proposed by Drapikowski and Domagala (16). An interactive segmentation model based on active shape was developed to extract the AAA tissues after manual segmentation of the first slice of the CT stack by de Bruijne et al. (17). A semi-automatic, interactive image segmentation model was also proposed by Maiora et al. (18) to detect lumen and ILT.

One recurring feature of published methods to segment the aortic wall is the need for some user intervention (semi-automated methods). Another commonality is the use of pre-processing steps, often used to define thresholds. These methods lack generality due to the possible inconsistencies between operators and the considerable geometrical and structural variability among different patients' aneurysms.

More recently, convolutional neural networks have been proposed for AAA segmentation from CT images (19–23). CNNs are very good feature extractors and good classifiers to discriminate between various extracted tissues, but they are not recommended for detecting and segmenting different tissues.

Finally, there are a few published examples of deep convolutional neural networks to segment the aorta, for example, the work by López-Linares et al. (24). In another recent study, thrombus segmentation using a region-based convolutional neural network was performed by Hwang et al. (25). A thrombus segmentation model followed by lumen segmentation using a U-net was proposed by Brutti et al. (26). Most of these recent studies approach the segmentation by directly segmenting the thrombus first. The ILT has a very irregular morphology and inconsistent tissue properties that vary considerably from patient to patient and even within one patient. Therefore, starting the segmentation directly by detecting the thrombus, results in training the model on very inconsistent features.

This study proposes a fully automatic segmentation model to segment the whole AAA. The model first detects the “aorta” as a whole, including the aortic wall, ILT, lumen, and calcification, and masks the original image to remove all surrounding similar organs and structures for more precise wall segmentation. This allows the subsequent networks to concentrate on identifying the wall, ILT, lumen, and calcification within an image that comprises only the aorta.

## 2. Materials and methods

**Figure 2:**
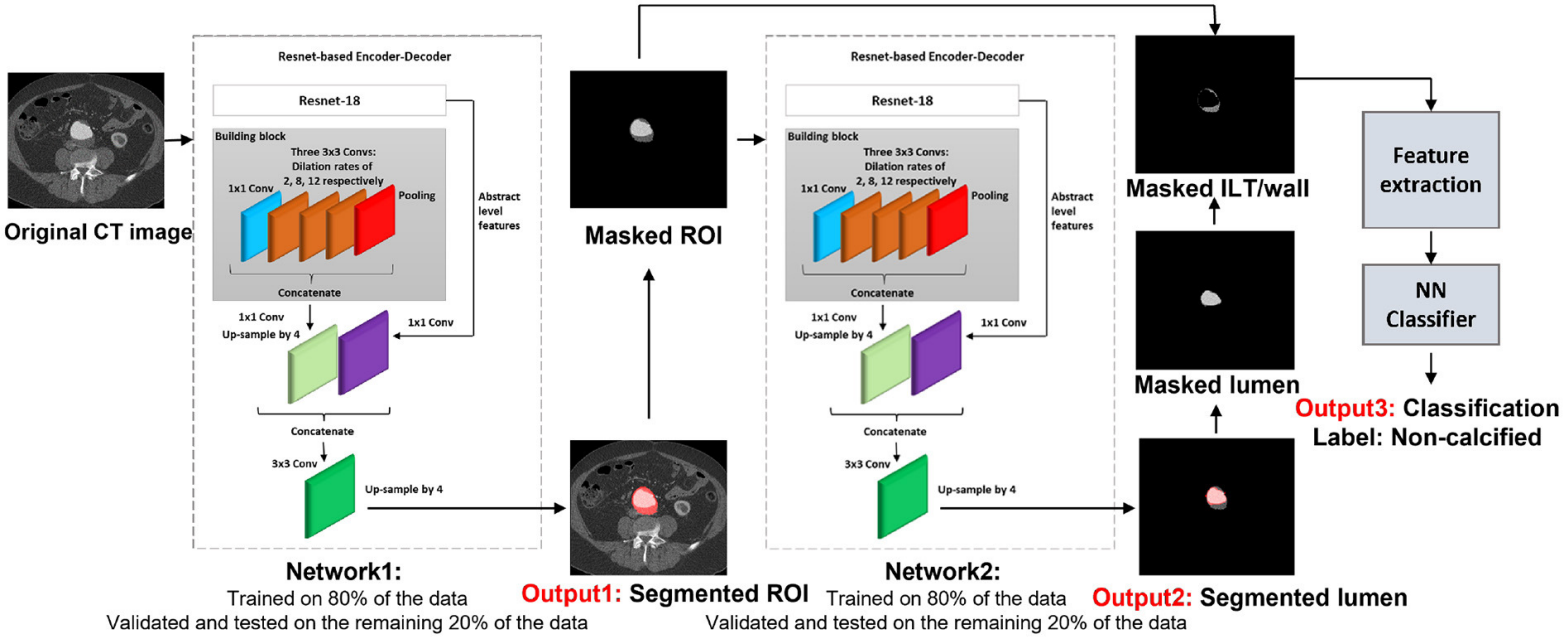
Visual representation of the network architecture and output for AAA tissue segmentation.

For the purpose of this article, the ROI was defined as the abdominal aorta including wall, lumen, ILT, and calcification from the celiac artery to the common iliac arteries, and the external and internal iliac arteries. The proposed model is composed of four trained networks. The first network receives the original CT image to detect and extract the ROI including the abdominal aorta and iliac arteries. Histogram equalization was performed on the input images to ensure that the trained model will be generalized to CT images from different CT scanners. The output of the first network is received by the second network to detect and extract the lumen. By extracting the lumen from the ROI, the remaining tissue is a combination of wall and ILT (ILT/wall). The third network receives the extracted ILT/wall and categorizes it slice-by-slice as calcified if any calcification is detected. Otherwise, the ILT/wall is categorized as non-calcified (Figure 2). A final fourth network was trained separately for landmark detection. This network receives the original CT image to detect and extract the ROI including the abdominal aorta, iliac, celiac, and renal arteries. The celiac and renal arteries are considered landmarks. The model architecture and segmentation steps are shown in Figure 2.

Experiments were performed according to the following steps: 1. Designing the model. 2. Fine-tuning and transfer learning to adapt a pre-trained network to our segmentation problem. 3. Training and validation of the networks at each step of the model. 4. Evaluating the performance of the final trained model on a set of 19 new patients that were not involved in any of the training, validation, and test sets. 5. Selecting two challenging cases and evaluating the performance of the final model on challenging cases. 6. Evaluating the performance of the proposed segmentation model on an external cohort of patients from a different institution. All the experiments were performed in MATLAB R2021b.

### 2.1. Data collection

The experiments were performed on 6030 CT slices from abdominal CT scans obtained from 56 different patients with AAA. Image acquisition was performed at the Peter Lougheed Centre in Calgary, Alberta, Canada.

The studies involving human participants were reviewed and approved by the Conjoint Health Research Ethics Board (CHREB), University of Calgary. Written informed consent to participate in this study was provided by the patient/participants.

The imaging protocol consisted of retrospectively gated multi-phase CT angiography (64-row helical GE Medical System CT scanner) with variable radiation dose to capture the R-R interval, with spatial resolution of approximately 0.7 × 0.7 × 2.0 mm. Notably, some scans had lower or higher image resolution varying from 0.6 × 0.6 × 2 mm to 0.9 × 0.9 × 2 mm. Gantry rotation time was equal to 0.35 s. Inclusion criteria were patients with diagnosed AAA from 2016 to 2020, aged 18+, and no prior aortic surgery.

We evaluated the generalizability of the algorithm performance on CT images obtained from 19 different patients that were not included in any of the training, validation, and test sets. This dataset had the same inclusion criteria.

As an external validation, we evaluated the performance of our proposed segmentation model on CT images obtained from six AAA patients with imaging performed at a different center. The images were collected from Centre hospitalier de l'Université de Montréal (CHUM) using TOSHIBA CT scanner with a spatial resolution of 0.8 x 0.8 x 0.8 mm.

Due to the retrospective nature of the study, we have demographic information only on a subset of 47 patients. The mean age±standard deviation is 77.4 ± 8.0. Also, five out of 47 patients are female.

All data were annotated by an expert operator using the commercial segmentation software Simpleware (Simpleware ScanIP, R-2020.09). On a subset of 10 patients, the manual annotations were validated pixel-by-pixel by an experienced vascular radiologist. The segmentation accuracy was deemed acceptable by the radiologist to be used as ground truth. To further verify the quality of the expert operator segmentation, an additional operator was trained, and inter-operator variability was quantified by comparing the masks obtained by the two on 19 patients.

### 2.2. Detection and extraction of the ROI

Inspired by the study of Chen et al. (27), we employed a Resnet-based fully convolutional network (FCN) with dilated convolutions. This network, an encoder–decoder with dilated convolution based on ResNet-18 architecture, was used as a pre-trained network. The model was developed in two steps: design of the model and transfer learning. First, we trained the model in different steps by detecting the ROI, which is the whole aortic structure including a combination of the aortic wall, ILT, lumen, and calcification. This way, the model searches for a combination of the aortic wall, lumen, ILT, and calcification as an extra feature, which helps remove all surrounding structures and organs with almost the same gray-scale level as the aortic wall. As a consequence, in the next steps, all other tissues including ILT, wall, and lumen can be segmented more accurately. Transfer learning and fine-tuning were performed by initializing the weights of the networks at each step by the weights of the pre-trained network and finding the optimal learning parameters. Each network is composed of stacked complex building blocks, each consisting of a combination of convolutional layers with kernel sizes of 1×1 and 3×3. The output features from each building block are concatenated into a single vector, which is the input of the next block. 1×1 convolutions were used for dimensionality reduction. The factorization of convolutions into small convolutions reduced the number of parameters as well as the computational cost while maintaining high efficiency. Dilated convolutions were used instead of standard convolutions for a larger field of view with the same computational cost, stride, and number of parameters as the standard convolution. This resulted in denser output features and higher segmentation performance. Dilated convolution was applied as follows:


(1) $$\begin{array}{l} y\left[i\right]=\sum_{k}x\left[i+r.k\right]w\left(k\right) \end{array}$$

where *x* is the input feature map and *y* is the output feature map. Dilated convolution was applied with a convolutional filter *w* over the input feature map *x* at each location *i* of the output feature map *y* for dimensionality reduction and providing a larger field of view.

We started fine-tuning from deeper network layers using grid searching for an extensive interval of values. Upper layers in the network architecture are responsible to extract more generic features of the images such as edges, borders, and shapes, which are common attributes in various applications. Caution was taken to allay over-fitting concerns in consideration of our small patient data set. The weights of all other layers remained constant by forcing the learning rates to zero for those layers. The learning process was performed using Adam as the model optimization algorithm. The optimization algorithm is used to minimize the loss function. The most important learning parameter that controls the adjustment of the model according to the updated weights at each iteration is the learning rate. The scheduling rate controls the learning rate at the end of each epoch according to the behavior of the learning curves and model convergence. In fast model convergence, the scheduling rate can be decreased after a few epochs but in slow convergence, a larger scheduling rate is required. Momentum is another parameter to be considered. Momentum is responsible to control the step sizes, while the optimization algorithm is searching for the global minimum. Small values of momentum may result in sub-optimal results since the algorithm can reach a local minimum by taking a small searching step and incorrectly consider it as the global minimum. The optimal learning parameters were obtained by evaluating the model performance on the validation set for each assigned value. The optimal learning parameter was determined to be 0.02. The momentum and scheduling rate were assigned as 0.8 and 0.9 at each step of the fine-tuning. The dilation rate was assigned as 2 and 4 for the last two blocks. An up-sampling factor of 4 was assigned to the decoder to up-sample the encoder output. The output of the decoder was combined with the low-level features after applying 1 x 1 convolution. The ROI was labeled as the first class and all the other surrounding tissues and the image background were labeled as the second class. Since the ROI is a small fraction of the whole image, we considered weighted loss functions. The performance of the network was evaluated by using both weighted cross-entropy and weighted generalized dice as loss functions. Weighted cross-entropy demonstrated better performance with the weight defined as follows:


(2) $$\begin{array}{l} w=\left(N-\sum_{n}p_{n}\right)/\sum_{n}p_{n} \end{array}$$

where *N* is the number of images annotated as foreground with predicted probabilistic map elements *p*_*n*_. Adam network optimizer was applied with L2 regularization of 0.0005, mini-batch size of 8, and validation patience of 6. The dataset was split into training, validation, and test sets. A total of 80% of the data was randomly selected for training, and the remaining 20% was split into two for validation and test sets. To avoid any bias, we ensured that there was no overlap between the three datasets. To validate the performance of the model on new patients' data, leave-one-out cross-validation was performed by leaving one patient data as the validation set and training the model on the data of all the other patients. This process was repeated 32 times for a subset of 32 different patients used in this study. To evaluate the results, at each step of the work, we measured the per-class accuracy, sensitivity, specificity, BF-score, and intersection-over-union (IoU) score based on the obtained confusion matrix as follows:


(3) $$\begin{array}{l} Accuracy=\frac{TP+TN}{TP+TN+FP+FN} \end{array}$$

(4) $$\begin{array}{l} Sensitivity=\frac{TP}{TP+FN} \end{array}$$

(5) $$\begin{array}{l} Specificity=\frac{TN}{TN+FP} \end{array}$$

(6) $$\begin{array}{l} Precision=\frac{TP}{TP+FP} \end{array}$$

(7) $$\begin{array}{l} BFscore=\frac{2\times Precision\times Sensitivity}{Precision+Sensitivity} \end{array}$$

(8) $$\begin{array}{l} IoUscore=\frac{TP}{TP+FP+FN} \end{array}$$

where TP, FP, FN, and TN are true positive, false positive, false negative, and true negative, respectively, for each patient.

### 2.3. Lumen, wall, and calcification detection

In this step, we segmented and extracted the lumen from the ROI. By extracting the lumen from the ROI, the remaining tissues are a combination of ILT and wall (ILT/wall). The same configuration of Resnet-based FCN was adapted in this step for lumen segmentation. The output of the previous step (extracted ROI including the whole aortic structure) was fed into the network for further processing to detect and extract the lumen. The lumen and the image background were labeled as the foreground and background, respectively, to train the model. A total of 80% of the data was used as the training set, and the remaining 20% was split into the validation and test sets. The results were validated by measuring the per-class accuracy, sensitivity, specificity, BF-score, and IoU-score using the obtained confusion matrix. Leave-one-out cross-validation was performed, and the process was repeated 32 times for a sub-set of 32 different patients used in this study.

In the next step, we evaluated the extracted ILT/wall to discriminate between calcified and non-calcified regions. Since calcification may not occur in all images, it is not efficient to train an FCN with a small number of images to detect calcification. With the existing dataset, we chose to discriminate between calcified and non-calcified ILT/walls by extracting deep features from all the extracted ILT/walls and training a classifier to consider the similarity between deep features and classify the calcified vs. non-calcified slices. Deep features are apt to describe various tissues and are strong discriminators. In CNN, the defined filter at each convolutional layer is responsible to move along the whole image with a defined stride and create a feature map. The upper layers of the network are responsible to extract abstract-level image information such as borders, shapes, and corners, while deeper layers are responsible to extract detailed image information such as complex texture features. A combination of a CNN as a feature extractor and a feed forward neural network as the classifier was applied. All the extracted ILT/walls were labeled manually as calcified or non-calcified. To be consistent in using the networks, we used features from the ILT/walls that were detected and extracted in the previous step. The extracted deep features were fed to a feed-forward neural network with 479 hidden layer neurons, which acts as the classifier. To find the optimal hidden size for the network, we evaluated the performance of the network for an extensive interval of hidden size values from 100 to 500. The training process was based on the scaled conjugate gradient method, while the parameter Sigma estimates the weight change for the second derivative approximation. To obtain the optimal value of Sigma, the performance of the classifier was evaluated by assigning various values from 0.0001 to 0.01. The highest performance of the network was obtained for the value of 0.085. The training was performed for 1,153 epochs, with a maximum validation failure of 191. For the number of epochs and validation failures, the performance of the network was evaluated for values ranging from 1 to 2,500 and 0 to 500, respectively.

### 2.4. Landmark detection

For added generality, we decided to perform the landmark detection separately from the rest of the structures in the aorta. The location of celiac and iliac arteries were considered as landmarks. Automated segmentation lends itself to be used successfully in accurately determining changes from baseline to follow-ups if appropriate landmarks are identified and labeled to ensure that the same segments of the aorta are evaluated for both baseline and follow-up. In addition, the renal artery was also included as a landmark because it is used by clinicians when planning surgical repair. To detect landmarks, the same configuration of the first network was trained to segment the aorta, iliac, celiac, and renal arteries as the ROI. Eighty percent of the data was selected randomly as a training set, and the remaining 20% of data was split equally into validation and test sets. We ensured that there was no overlap between any of the training, validation, and test sets. To evaluate the landmark network performance, the output of the network was compared against the ground truth by measuring accuracy, sensitivity, specificity, BF-score, and IoU-score on the test set.

## 3. Results

**Figure 3:**
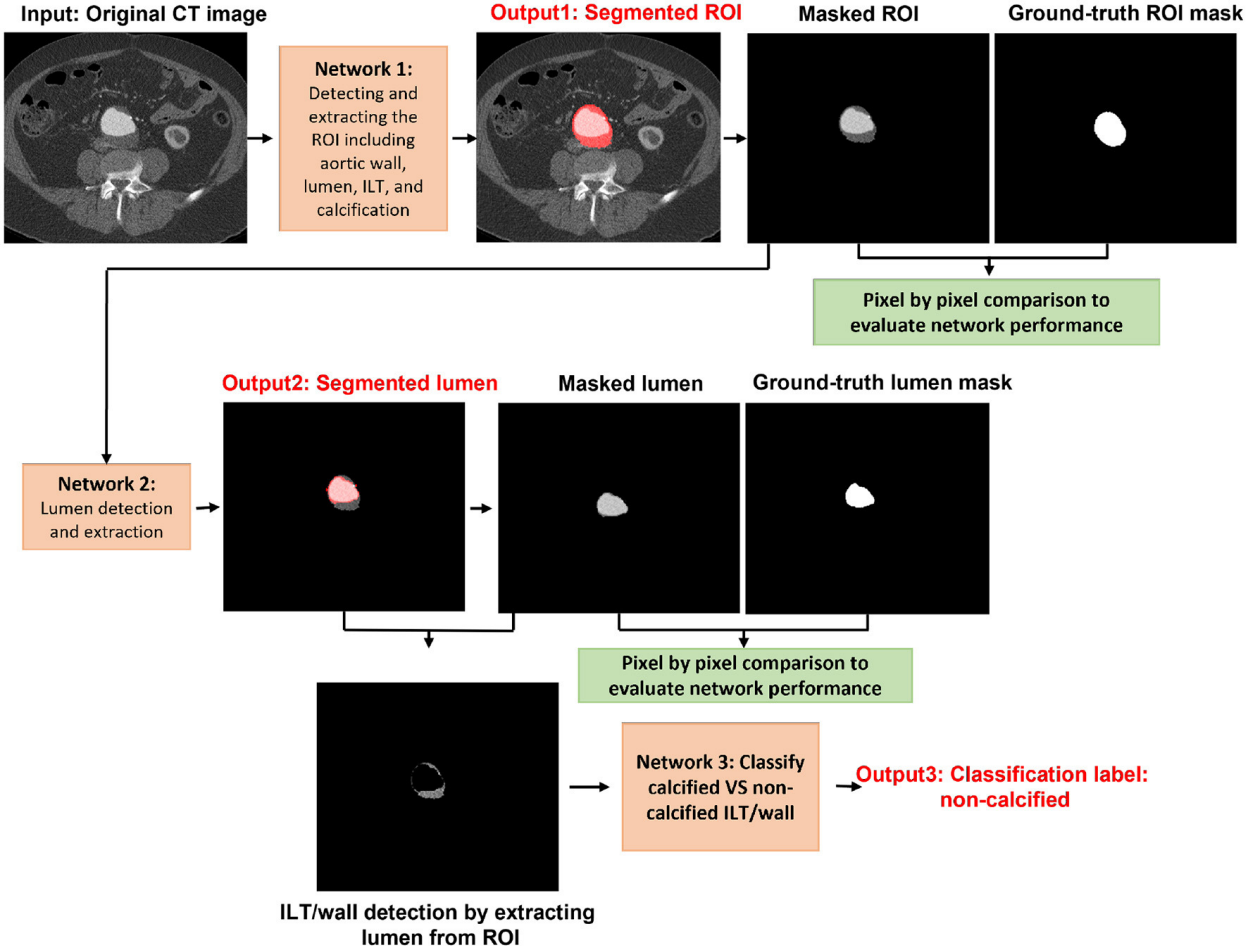
Visual representation of each network output for segmenting the ROI including the abdominal aorta, lumen, ILT, and wall. Calcification accumulation in ILT and wall was determined in the final step.

In the first step of the model, we segmented and extracted the ROI including the abdominal aorta and iliac arteries using a Resnet-based FCN (Figure 3). The measured per-class accuracy, sensitivity, specificity, BF-score, and IoU-score for the extraction of the ROI are shown in Table 1. Leave-one-out cross-validation was performed by leaving one patient data as the validation set and training the model on the data of all the other patients. The measured accuracy over all 32 patients was obtained as 0.94 ± 0.04. A network with the same configuration was trained in the second step to detect the lumen from the extracted ROIs (Figure 3). The measured accuracy, sensitivity, specificity, BF-score, and IoU-score for lumen extraction are shown in Table 1. At this step, leave-one-out cross-validation was performed, for a sub-set of 32 different patients used in this study. The measured accuracy over all 32 patients was obtained as 0.95 ± 0.03. Finally, a neural network was trained to classify the ILT/walls as calcified or non-calcified (Figure 3). The results are shown in Table 2.

**Table 1:** Measured accuracy, sensitivity, specificity, BF-score, and IoU-score to evaluate the performance of the segmentation model.

**Tissues under review**	**Accuracy**	**Sensitivity**	**Specificity**	**BF-Score**	**IoU-Score**
Aorta	0.99 ± 0.01	0.98 ± 0.02	0.99 ± 0.01	0.97 ± 0.03	0.98 ± 0.02
Lumen	0.98 ± 0.01	0.97 ± 0.03	0.99 ± 0.01	0.99 ± 0.01	0.97 ± 0.03
Landmark	0.99 ± 0.01	0.97 ± 0.03	0.99 ± 0.01	0.98 ± 0.02	0.97 ± 0.03

**Table 2:** Measured accuracy, sensitivity, and specificity for the classification of calcified vs. non-calcified ILT/wall.

**Tissues under review**	**Accuracy**	**Sensitivity**	**Specificity**
Calcified ILT/wall	0.91	0.91	0.90
Non-calcified ILT/wall	0.85	0.81	0.90

**Figure 4:**
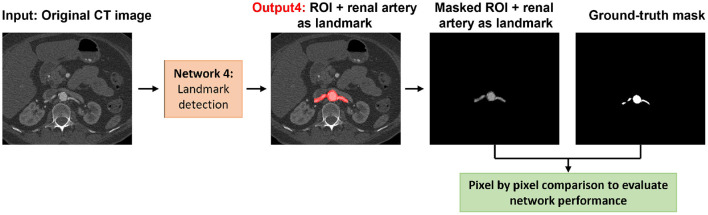
Visual representation of the landmark detection including the detection of the aorta, iliac, celiac, and renal arteries.

**Figure 5:**
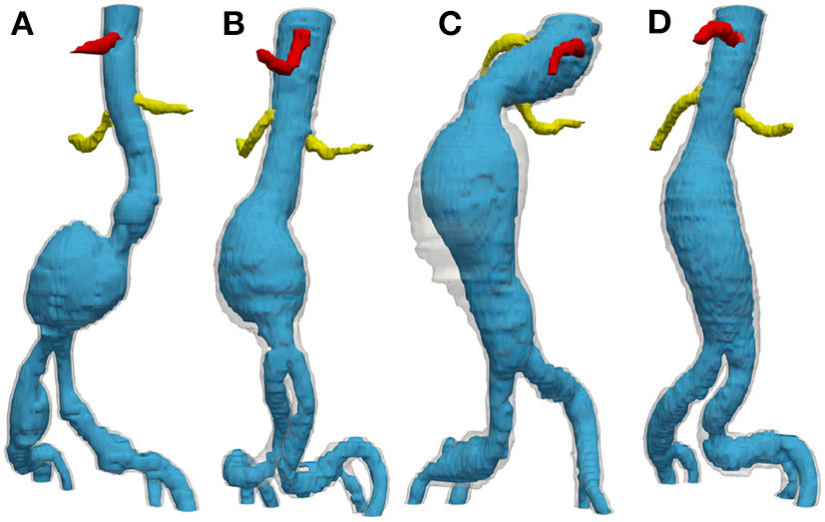
3D reconstruction obtained from the results of the automatic segmentation model for four different patients **(A–D)**. For each patient, the lumen is visualized in blue, the aortic ILT/wall is transparent, while the celiac and renal arteries are shown in red and yellow, respectively.

The results of landmark detection are shown in Table 1 and Figure 4. The final 3D reconstruction was performed in Simpleware (Synopsis) following the extracted ROIs for four different patients using our proposed automatic segmentation algorithm (Figure 5) only to visualize the results of our proposed segmentation model in 3D.

After preparing the network architecture and finding the optimal parameters and training options, the performance of the final trained networks was evaluated on 19 different patients with AAA. These patients were never introduced to the trained networks. They were not included in any of the training, test, or validation sets and were used solely to evaluate the generalizability of the proposed model. Measured accuracy, sensitivity, specificity, BF-score, and IoU-score of all 19 patients are shown in Table 3. In these 19 patients, we selected two challenging cases to report the model performance. Patients 1 and 2 were considered challenging cases. Patient 1 presented a very tortuous aorta. This tortuosity created a visual artifact in the axial CT acquisition and resulted in a select number of images presenting two lumens in the middle portion of the aorta. The iliac bifurcation for patient 2 also presented some tortuosity, with the two iliac arteries developing mostly in a horizontal direction with respect to the acquisition plane instead of the longitudinal direction. Therefore, the iliac arteries of this patient did not appear as circular but as structures elongated in the acquisition plane. The CT images of these two challenging cases were used as the input of each trained network. The mean and standard deviation of the accuracy, sensitivity, specificity, BF-score, and IoU-score were measured for each patient separately. The results are shown in Tables 4–6.

**Table 3:** Measured accuracy, sensitivity, specificity, BF-score, and IoU-score to evaluate the model performance on 19 different patients, which were not included in any of the training, test, and validation sets and were only used for algorithm verification.

**Tissues under review**	**Accuracy**	**Sensitivity**	**Specificity**	**BF-Score**	**IoU-Score**
Aorta	0.94 ± 0.06	0.88 ± 0.09	0.99 ± 0.01	0.92 ± 0.08	0.88 ± 0.09
Lumen	0.96 ± 0.04	0.92 ± 0.08	0.99 ± 0.01	0.93 ± 0.07	0.92 ± 0.08
Landmark	0.92 ± 0.06	0.84 ± 0.10	0.99 ± 0.01	0.86 ± 0.09	0.84 ± 0.09

We reported mean (±) std of measured accuracies, sensitivities, specificities, BF-scores, and IoU-scores in all 19 patients.

**Table 4:** Measured accuracy, sensitivity, specificity, BF-score, and IoU-score for the detection of the aorta in CT images of the two challenging cases.

**Patients**	**Aorta accuracy**	**Aorta sensitivity**	**Aorta specificity**	**Aorta BF-score**	**Aorta IoU-score**
Patient1	0.98 ± 0.02	0.95 ± 0.05	0.99 ± 0.01	0.92 ± 0.08	0.95 ± 0.05
Patient2	0.96 ± 0.04	0.91 ± 0.09	0.99 ± 0.01	0.89 ± 0.09	0.91 ± 0.09

**Table 5:** Measured accuracy, sensitivity, specificity, BF-score, and IoU-score for lumen detection in CT images of the two challenging cases.

**Patients**	**Lumen accuracy**	**Lumen sensitivity**	**Lumen specificity**	**Lumen BF-score**	**Lumen IoU-score**
Patient1	0.99 ± 0.01	0.98 ± 0.02	0.99 ± 0.01	0.92 ± 0.08	0.98 ± 0.02
Patient2	0.96 ± 0.04	0.93 ± 0.07	0.99 ± 0.01	0.92 ± 0.08	0.93 ± 0.07

**Table 6:** Measured accuracy, sensitivity, specificity, BF-score, and IoU-score for the landmark detection including the aorta, iliac, celiac, and renal arteries on CT images of the two challenging cases.

**Patients**	**Landmark** **accuracy**	**Landmark** **sensitivity**	**Landmark** **specificity**	**Landmark** **BF-score**	**Landmark** **IoU-score**
Patient1	0.97 ± 0.03	0.95 ± 0.05	0.99 ± 0.01	0.93 ± 0.07	0.95 ± 0.05
Patient2	0.96 ± 0.04	0.91 ± 0.09	0.99 ± 0.01	0.93 ± 0.07	0.91 ± 0.09

We also evaluated the performance of the segmentation model on an external cohort. The images were collected from a different center and CT scanner that were never introduced to the network and were not included in any of the training, test, or validation sets. The results are shown in Table 7.

**Table 7:** Measured accuracy, sensitivity, specificity, BF-score, and IoU-score to evaluate the model performance on an external cohort of six different patients that were not included in any of the training, test, and validation sets and were only used for algorithm verification.

**Tissues under review**	**Accuracy**	**Sensitivity**	**Specificity**	**BF-Score**	**IoU-Score**
Aorta	0.94 ± 0.06	0.88 ± 0.09	0.99 ± 0.01	0.88 ± 0.10	0.88 ± 0.09
Lumen	0.98 ± 0.02	0.96 ± 0.03	0.99 ± 0.01	0.91 ± 0.09	0.96 ± 0.04
Landmark	0.94 ± 0.05	0.88 ± 0.10	0.99 ± 0.01	0.90 ± 0.09	0.88 ± 0.10

We reported the mean (±) std of measured accuracies, sensitivities, specificities, BF-scores, and IoU-scores in all six patients.

Results of inter-operator variability are shown in Table 8. This comparison was performed by comparing the masks created by two expert operators.

**Table 8:** Inter-operator variability by comparison the segmentation masks created by two expert operators.

**Operators**	**Tissues under review**	**Accuracy**	**Sensitivity**	**Specificity**	**BF-Score**	**IoU-Score**
Operator1 VS Operator2	Aorta	0.98 ± 0.02	0.95 ± 0.05	0.99 ± 0.01	0.98 ± 0.02	0.95 ± 0.05
	Lumen	0.94 ± 0.05	0.88 ± 0.10	0.99 ± 0.01	0.98 ± 0.02	0.88 ± 0.11
	Landmark	0.97 ± 0.03	0.94 ± 0.06	0.99 ± 0.01	0.97 ± 0.02	0.94 ± 0.04

The results are reported as mean (±) std of all the measured accuracy, sensitivity, specificity, BF-score, and IoU-score for the CT images of all 19 patients.

## 4. Discussion

In this study, we proposed a fully automatic model to segment the whole AAA. The following considerations were taken into account in designing the model to improve the existing limitations of recent studies. Most of the recent articles started by training a deep learning model to segment and extract the ILT/wall as a first step (24–26). The ILT is a complex tissue with highly inconsistent properties. It is highly variable among patients not only as a result of patient variability but also as a result of the stage of disease progression and thrombus formation. Furthermore, within the same patient we may have ILT exhibiting different features (more or less porous, more or less calcified) depending on how the ILT is responding and adapting to the regional environment and also depending on the deposition and formation stage of the thrombus. In addition, the aorta is a small subset of an abdominal CT scan image and many of the structures in view have similar intensities. For this reason, the direct segmentation of the ILT/wall is challenging and increases the need to include pre-processing steps. We overcame this limitation by proposing a model which segments and extracts the aorta including ILT/wall and lumen in two main steps: 1. Segmenting and extracting the whole aortic structure as the ROI in the first step. To achieve this, in addition to the extracted deep features from the ROI, we introduced an extra feature to the network to look for the whole aortic structure as a combination of the aortic wall, ILT, lumen, and calcification. The early detection and extraction of the ROI removes all the surrounding organs and structures resulting in precise and accurate detection of the ILT/wall and lumen in the next step. 2. Training the second network to receive the extracted ROI as the input and recognize the lumen in the ROI. Once the lumen is segmented and extracted from the ROI, the remaining tissue is the ILT/wall.

The use of pre-processing steps for AAA images can lead to problems due to the variations in the background at different levels along the abdomen. Most pre-processing methods are based on filtering and defining specific thresholds, which cannot be generalized to a variety of CT acquisitions (8–11, 13, 19, 20, 22–24, 28, 29). Our only pre-processing step is histogram equalization to ensure that the proposed segmentation model can be applicable to different CT scanners.

One of the improvements of our approach with respect to many prior studies is that it does not employ patch-based segmentation using CNNs (19–24). In a CNN-patch-based segmentation, the patches will overlap in order to analyze all the pixels, resulting in a redundant feature extraction which is computationally expensive and time-consuming. Moreover, since the input size is fixed in CNNS, the patch size needs to be adapted to it.

Fully convolutional networks can overcome the limitations of patch-based segmentation using CNNs. FCNs can be trained on a smaller number of images with higher pixel-wise segmentation precision. In FCNs, the pooling operators are replaced by up-sampling operators to enhance the output resolution. An arbitrary image size can be fed to the network since there is no fully connected layer involved in the network architecture and the network is trained end-to-end, pixel-to-pixel to optimize the process for accurate segmentation. One of the clearest advantages is that extensive pre-processing is not necessary when using FCNs. Another advantage of our method with respect to existing FCN studies (24–26) is the automatic extraction of the ROI using semantic segmentation, which greatly simplified the subsequent steps of segmenting the structures inside the aorta.

One of the main limitations of this study is the use of 2D segmentation, which may cause inconsistencies between two adjacent slices. To address the limitations of our proposed model, we plan on implementing a 3D version of the networks, once a sufficient number of patients is available. Future improvements can also be achieved by training on more data from different CT scanners and different institutions.

A possible future advantage of using a fully automatic segmentation model is to eliminate the need for a contrast agent during CT angiography imaging since the detection of the AAA tissues is not done visually. The automatic model can detect various tissues by recognizing their features including shape, borders, and detailed texture instead of relying mainly on differences in pixel intensities.

## 5. Conclusion

This study was focused on developing an automatic deep learning-based segmentation model for the segmentation of the whole AAA, including the wall, lumen, ILT, and calcification in the aorta and iliac arteries. The proposed model overcomes many existing limitations of automated segmentation models by introducing a first step of detecting and extracting the full aortic structure from the image. Future studies will focus on extending the fully automatic segmentation model to cover other aortic diseases including thoracic AA and dissections.

## 6. Patent

A PCT entitled “METHOD AND SYSTEM FOR SEGMENTING AND CHARACTERIZING AORTIC TISSUES” was filed with the international application number PCT/IB2022/051558.

## Data availability statement

The datasets presented in this article are not readily available because this dataset is confidential. Requests to access the datasets should be directed to elenadimartino@vitaamedical.com.

## Ethics statement

The studies involving human participants were reviewed and approved by Conjoint Health Research Ethics Board (CHREB), University of Calgary. The patients/participants provided their written informed consent to participate in this study. Written informed consent was obtained from the individual(s) for the publication of any potentially identifiable images or data included in this article.

## Author contributions

AA: conceptualization, investigation, methodology, software, validation, writing—original draft, and visualization. AF: data curation, ground-truth creation, conceptualization, and writing—review and editing. RM: investigation, patient recruitment, and writing—review and editing. ED: conceptualization, supervision, and writing—review and editing. All authors contributed to the article and approved the submitted version.
